# Transcriptional dysregulation of Interferome in experimental and human Multiple Sclerosis

**DOI:** 10.1038/s41598-017-09286-y

**Published:** 2017-08-21

**Authors:** Sundararajan Srinivasan, Martina Severa, Fabiana Rizzo, Ramesh Menon, Elena Brini, Rosella Mechelli, Vittorio Martinelli, Paul Hertzog, Marco Salvetti, Roberto Furlan, Gianvito Martino, Giancarlo Comi, Eliana M. Coccia, Cinthia Farina

**Affiliations:** 1 Institute of Experimental Neurology (INSpe), Division of Neuroscience, San Raffaele Scientific Institute, 20132 Milan, Italy; 2 University Vita-Salute San Raffaele, 20132 Milan, Italy; 3 Department of Infectious diseases, Istituto Superiore di Sanità, 00161 Rome, Italy; 4 University “La Sapienza” Rome, Dept. Neurological Sciences, MS center of S. Andrea Hospital, 00185 Rome, Italy; 5 Centre for Innate Immunity and Infectious Diseases, Hudson Institute of Medical Research, Monash University, 3168 Clayton, IS Australia; 6 IRCCS Istituto Neurologico Mediterraneo (INM) Neuromed, 86077 Pozzilli, IS Italy

## Abstract

Recent evidence indicates that single multiple sclerosis (MS) susceptibility genes involved in interferon (IFN) signaling display altered transcript levels in peripheral blood of untreated MS subjects, suggesting that responsiveness to endogenous IFN is dysregulated during neuroinflammation. To prove this hypothesis we exploited the systematic collection of IFN regulated genes (IRG) provided by the Interferome database and mapped Interferome changes in experimental and human MS. Indeed, central nervous system tissue and encephalitogenic CD4 T cells during experimental autoimmune encephalomyelitis were characterized by massive changes in Interferome transcription. Further, the analysis of almost 500 human blood transcriptomes showed that (i) several IRG changed expression at distinct MS stages with a core of 21 transcripts concordantly dysregulated in all MS forms compared with healthy subjects; (ii) 100 differentially expressed IRG were validated in independent case-control cohorts; and (iii) 53 out of 100 dysregulated IRG were targeted by IFN-beta treatment *in vivo*. Finally, *ex vivo* and *in vitro* experiments established that IFN-beta administration modulated expression of two IRG, ARRB1 and CHP1, in immune cells. Our study confirms the impairment of Interferome in experimental and human MS, and describes IRG signatures at distinct disease stages which can represent novel therapeutic targets in MS.

## Introduction

Multiple sclerosis (MS) is a chronic inflammatory demyelinating disorder of the central nervous system (CNS) presenting with unpredictable and heterogeneous clinical courses. The first clinical occurrence of neurological dysfunction with features suggestive of a demyelinating inflammatory event is termed clinically isolated syndrome (CIS)^1^. The most frequent clinical course of MS, the relapsing-remitting form (RR-MS), is characterized by periodic worsening of the neurological condition followed by partial or complete remission. After several years most of the RR-MS patients develop the secondary progressive course (SP-MS) with advancing neurological dysfunction without recognizable relapses^1^. In a small fraction of MS subjects the progressive form of the disease is seen from onset and is referred as primary progressive (PP-MS). MS is a complex disorder where several genetic and environmental factors are involved^2^. The most recent high-throughput genetic screening in MS has highlighted that many genetic risk variants are located in the vicinity of immunologically relevant genes^3^. Interestingly these genes include several IRG, like TYK2, OAS1, STAT3 and STAT4, suggesting the genetically determined dysfunction of the pathways regulated by interferons in MS. Interferons (IFN) are a major class of proteins released by the host in response to pathogens, with IFN-beta being involved in antiviral defense^4^. Notably, the administration of recombinant IFN-beta is a first-line treatment for RR-MS^5^, indicating that the stimulation of that pathway *in vivo* is beneficial to MS patients. Our hypothesis is that MS is characterized by dysregulation of the genes involved in IFN signaling. To demonstrate it we (1) recovered IRG from the Interferome database, which collects and analyzes publicly available gene expression experiments where cells were exposed to IFN, (2) systematically checked their expression and enrichment in immune cells and/or CNS of human or experimental MS, and (3) verified their modulation by IFN-beta *in vivo* and *in vitro*.

## Results

### Transcriptomic analysis of Interferome in experimental MS

**Figure 1:**
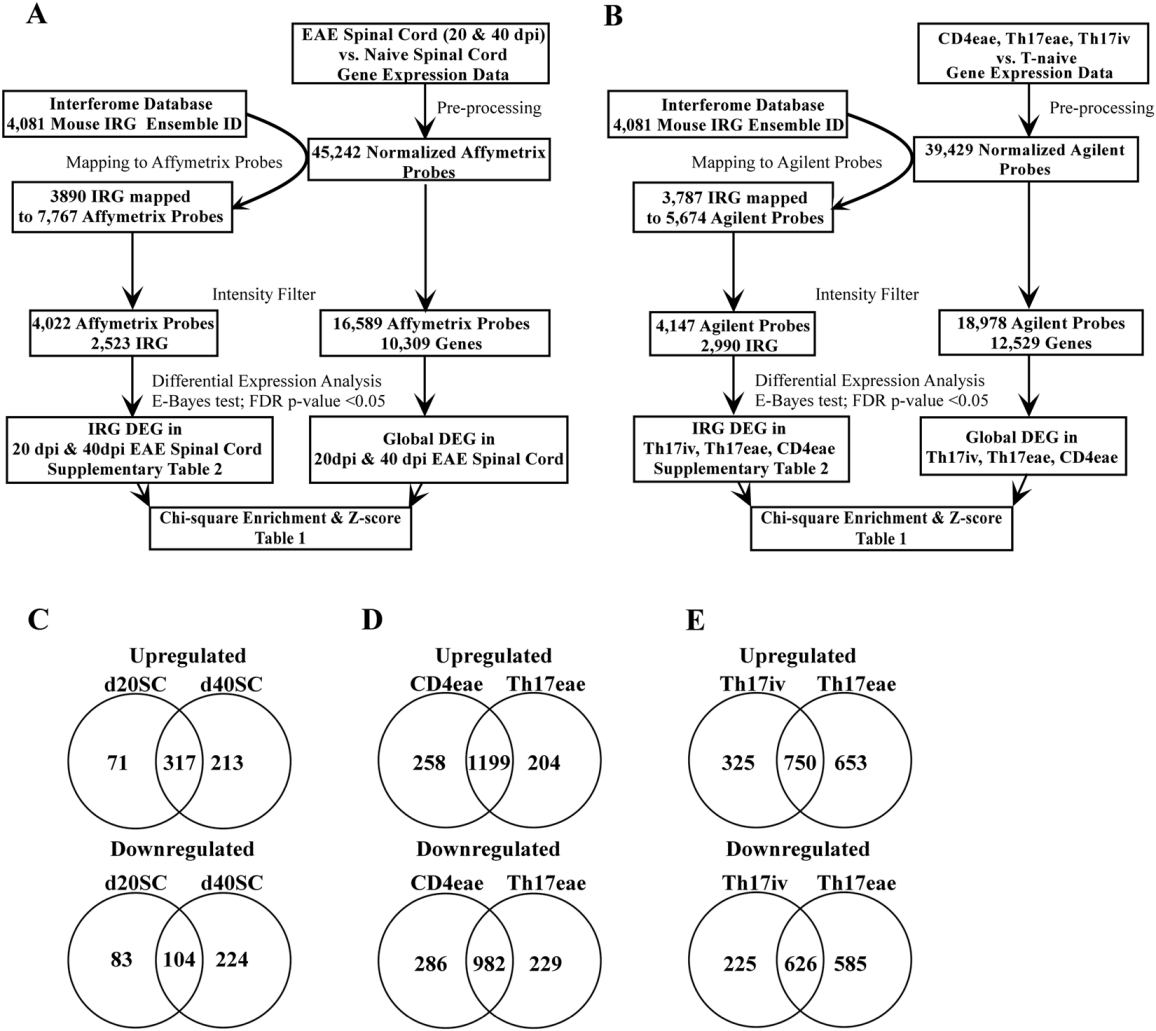
(**A**) Flow chart for the transcriptomics study of mouse spinal cord. (**B**) Flow chart for the transcriptomics study of mouse CD4 T lymphocytes. (**C**) Number of common and unique dysregulated IRG in EAE spinal cord (SC) at day 20 and 40 post immunization. (**D**) Number of common and unique dysregulated IRG in CD4eae and Th17eae T lymphocytes. (**E**) Number of common and unique IRG DEG in Th17iv and Th17eae T lymphocytes.

To investigate the expression levels of the genes regulated by interferons during experimental neuroinflammation, we performed high-throughput transcriptomic profiling of spinal cord tissues isolated from EAE mice at day 20 and 40 post-immunization (dpi) and age-matched naïve control animals. At the same time we queried Interferome, a manually curated, systematic collection of IRG identified in published transcriptomics studies, to retrieve 4081 mouse IRG, which were then mapped to the corresponding Affymetrix probes using biomaRt ID conversion package in R Bioconductor (see workflow in Fig. 1A). After preprocessing and intensity filter, we examined whether 4022 Affymetrix probes corresponding to 2523 IRG were differentially expressed in the spinal cord of EAE mice at 20 and 40 dpi compared to control samples from naïve mice. A total of 575 and 858 IRG probes was dysregulated at 20 and 40 dpi respectively (Supplementary Table 2) with most of the IRG up-or down-regulated in EAE mice at 20 dpi appearing also at 40 dpi (Fig. 1C), including the upregulation of MHC molecules, chemotactic mediators (CXCL10, CCL5, CCL6, CXCL12), typical IFN-induced proteins (GBP proteins) and transcription factors (IFN regulatory transcription factor (IRF)8, IRF9, signal transducer and activator of transcription (STAT)1, STAT3). To verify whether Interferome elements were enriched in the CNS transcriptome of EAE animals, we measured the frequency of differential expression (DE) among IRG and compared it to the frequency of dysregulation in the global transcriptome by chi-square and z-score statistics. We found that the frequency of dysregulation in IRG in the CNS of EAE animals at 20 and 40 dpi was significantly higher than the expected frequency of dysregulation in a random selection of transcripts (Table 1).

**Table 1:** Probability of IRG DEG enrichment in CNS and CD4 T lymphocytes during experimental neuroinflammation.

	IRG DEG	All IRG	IRG DEG %	Global DEG	Expressed Global genes	Global DEG %	Chi square (Yate’s correction)	P-value	Z-score
D20_Sc	575	4022	14.29	1052	16589	6.34	563.9	0.00001	23.7
D40_Sc	858	4022	21.33	2983	16589	17.98	40.12	0.00001	6.35
Th17iv	1926	4147	46.44	7704	19149	40.2	84.60	0.00001	9.21
Th17eae	2614	4147	63.03	10556	19014	55.5	120.9	0.00001	11.01
CD4eae	2725	4147	65.71	11698	18978	61.6	36.96	0.00001	6.09

DEG = differentially expressed gene; Sc = Spinal cord; eae = experimental autoimmune encephalomyelitis; Th17iv = *in vitro* differentiated T helper 17 cells.

Next, we investigated the expression levels of Interferome specifically in mouse CD4^+^ T cells during EAE or *in vitro* differentiation. The study by Hoppmann *et al*.^6^ generated transcriptomes from myelin-specific CD4+CD62L+T lymphocytes before (T naïve) and after *in vitro* Th17 differentiation (Th17 iv), myelin-reactive CD4+Th17 T lymphocytes isolated from the CNS of EAE mice after adoptive transfer (Th17eae), and CD4+T lymphocytes isolated from the CNS of EAE mice after active immunization (CD4eae)^6^. Thus, we retrieved the global transcriptome and isolated the expression values of IRG probes in T naïve, Th17iv, Th17eae, and CD4eae. As shown in Fig. 1B, there were 4147 Agilent probes corresponding to 2990 IRG passing the intensity filter criteria. Similarly to the CNS analysis, we searched for transcripts dysregulated in Th17iv, Th17eae, and CD4eae cells compared to naïve CD^4^+T cells (Supplementary Table 2), and found that the great majority of IRG altered in CD4^+^T cells isolated from the CNS of EAE mice were present also in Th17 T cells after adoptive transfer (Fig. 1D), indicating that Th17 transcriptome well replicates the transcriptional changes of all CD4 cells during EAE in this model. Several cytokines (IL6, IL1b, IL18), chemokines (CCL2, CCL3, CCL5, CXCL9, CXCL10), and interferon regulatory factors (IRF4, IRF8) appeared among the dysregulated IRG in CD4eae and Th17eae. *In vitro* T cell differentiation towards the Th17 phenotype was characterized by several changes in Interferome which were mostly reproduced by Th17 cells isolated from EAE mice (Fig. 1E). Similarly to the EAE CNS transcriptomic profiles, we observed a clear and significant enrichment of IRG DEG in transcriptomes of CD4^+^ T lymphocytes from EAE animals or after *in vitro* Th17 differentiation (Table 1). Overall, we found robust evidence of IRG dysregulation in both CNS and CD4^+^ T cells of experimental MS.

### Transcriptomic analysis of Interferome in MS

**Figure 2:**
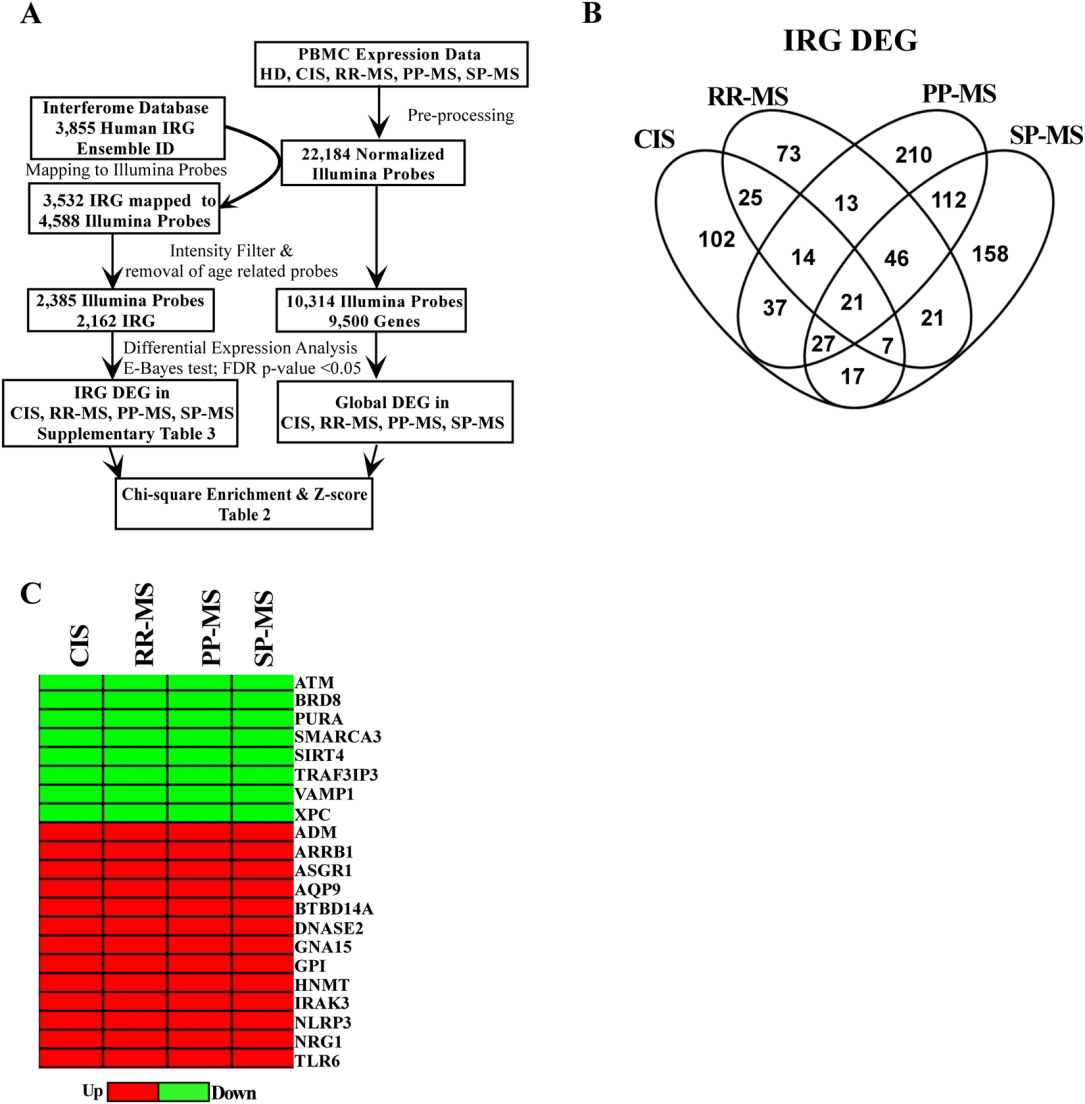
(**A**) Flow chart for the human PBMC transcriptomics study at distinct MS stages. (**B)** Number of common and unique IRG DEG for each disease stage. (**C**) Fold change heatmap representing significantly upregulated (red color) or down regulated (green color) IRG at distinct MS stages compared to healthy subjects.

**Figure 3:**
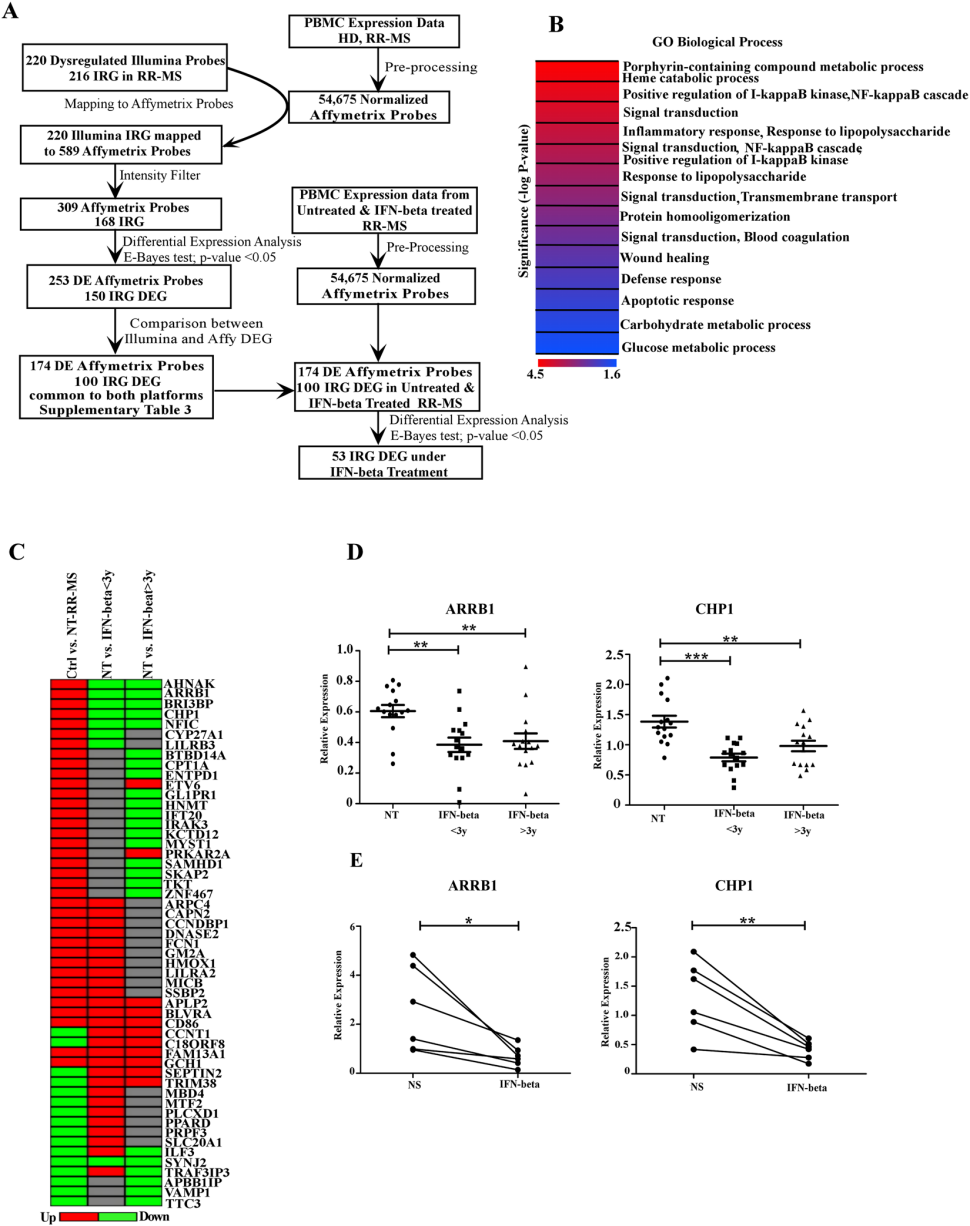
(**A**) Flow chart for the human PBMC transcriptomics studies in RR-MS. (**B**) Significant Gene Ontology terms relative to the 100 validated IRG DEG in RR-MS. (**C)** Fold change heatmap representing the dysregulated IRG at baseline in RR-MS (first column) which are targeted *in vivo* by IFN-beta treatment (second and third columns). (**D)** ARRB1 and CHP1 transcript levels in PBMC from a novel independent cohort of untreated (NT) RR-MS and IFN-beta treated RR-MS as detected by quantitative PCR. **p-value < 0.01, ***p-value < 0.001 (**E)** ARRB1 and CHP1 transcript levels in PBMC from RR-MS subjects stimulated *in vitro* in absence (NT) or presence of IFN-beta, *p-value < 0.05, **p-value < 0.01.

To verify dysregulation of Interferome in human MS, we retrieved human IRG from the online database^7^, mapped them to the relative array probe identifiers, isolated their expression levels from PBMC transcriptomes of CIS, RR-MS, PP-MS, SP-MS subjects and healthy individuals (Fig. 2A), and conducted differential expression analysis to evaluate whether the probes corresponding to IRG were dysregulated at distinct clinical phases of disease compared to healthy controls. After applying false discovery rate corrected P-value threshold <0.05, we defined 250 differentially expressed probes in CIS, 220 in RR-MS, 480 in PP-MS and 409 in SP-MS respectively (Supplementary Table 3), with several of them dysregulated in at least 2 disease forms (Fig. 2B). Notably, 21 IRG were dysregulated in all disease groups compared to the healthy population (Fig. 2B) and direction of dysregulation was conserved in all cases (Fig. 2C). Specifically, the transcripts for ATM, BRD8, PURA, SMARCA3, SIRT4, RAF3IP3, VAMP1, and XPC were downregulated, while those for ADM, ARRB1, ASGR1, AQP9, BTBD14A, CDKN2D, DNASE2, GNA15, GPI, HNMT, IRAK3, KIF1B, NLRP3, NRG1, and TLR6 were upregulated under disease compared to healthy subjects. To evaluate whether MS transcriptome was enriched in dysregulated IRG, we measured the frequency of differential expression in the list of expressed IRG and in the global transcriptome for each MS form (Table 2), and calculated whether the frequency of dysregulation of IRG was significantly higher in the IRG list than the expected frequency of differential regulation in a random selection of transcripts from the whole transcriptome. As shown in Table 2, IRG DEG enrichment was significant in RR-MS and not in the early phases of disease or the progressive MS population. Considering the high frequency of dysregulated Interferome in RR-MS, we further checked the expression of dysregulated IRG in PBMC of RR-MS in a distinct case-control cohort for which whole genome transcriptome was generated by Affymetrix arrays (Fig. 3A), and validated a total of 100 IRG DEG with concordant regulation compared to healthy subjects in both independent cohorts measured with distinct array platforms (Fig. 3A and Supplementary Table 3). Gene ontology analysis unraveled significant enrichment in genes playing a role in NFkB pathway, immune defense, and apoptosis (Fig. 3B). Overall, several IRG changed expression at distinct MS stages, part of them were concordantly regulated in all MS forms, but alterations in Interferome were significantly enriched only in RR-MS.

**Table 2:** Probability of IRG DEG enrichment at distinct stages of multiple sclerosis.

	IRG DEG	All IRG	IRG DEG %	Global DEG	Expressed Global genes	Global DEG %	Chi square (Yate’s correction)	P-value	Z-score
CIS	250	2385	10.48	1010	10314	9.79	1.570	0.2101	1.29
RR-MS	220	2385	9.22	764	10314	7.4	14.59	0.0001	3.86
PP-MS	480	2385	20.12	2039	10314	19.76	0.220	0.6388	0.49
SP-MS	409	2385	17.14	1854	10314	17.97	1.366	0.2425	−1.19

HC = Healthy controls; CIS = clinically isolated syndrome; RR-MS = Relapsing-Remitting multiple sclerosis; PP-MS = Primary progressive multiple sclerosis; SP-MS = Secondary progressive multiple sclerosis; DEG = differentially expressed genes.

### IFN-beta modulates dysregulated IRG in RR-MS

To check whether IFN-beta treatment modulates baseline dysregulation of Interferome in RR-MS, we recovered the expression values of the 100 validated IRG in RR-MS from a published dataset^8^ comprising PBMC transcriptomic profiles from 50 RR-MS subjects under IFN-beta treatment for less than three years, 43 RR-MS patients under IFN-beta treatment for more than 3 years and 141 untreated RR-MS subjects (Fig. 3A). Importantly, 53 of 100 dysregulated IRG at baseline were targeted by IFN-beta treatment (Fig. 3C and Supplementary Table 4), with some transcripts counter-regulated (e.g. ARRB1, CHP1, IRAK3) and others further sustained (e.g. APLP2, BLVRA, CD86) under IFN-beta treatment. Down regulation of ARRB1 and CHP1 upon IFN treatment was validated by quantitative real time PCR in independent cohorts of IFN-beta-treated and untreated RR-MS patients (Fig. 3D). Further, short *in vitro* exposure to IFN-beta was sufficient to downregulate ARRB1 and CHP1 expression in PBMC from untreated RR-MS subjects (Fig. 3E).

## Discussion

In this study we provide evidence for transcriptional dysregulation of Interferome in the spinal cord of experimental MS, in the CNS-infiltrating encephalitogenic mouse T lymphocytes and in the peripheral blood mononuclear cells of human subjects with distinct stages and clinical courses of multiple sclerosis.

We have recently reported the selective dysregulation of MS risk genes in peripheral blood of MS patients^9^. Interestingly, some of them play a role in IFN-mediated pathways, e.g. TYK2 and STAT4, and show aberrant expression in MS when compared to the healthy population, with TYK2 upregulated in PBMC from progressive MS and STAT4 downregulated in early and progressive stages of MS^9^. These data indicate that single risk genes involved in IFN signaling may be dysregulated in MS, and that this phenomenon is not necessarily conserved throughout all MS stages, raising the hypothesis that Interferome may be characterized by transcriptomic changes specific to disease type. Among several reports indicating transcriptional dysregulation induced by IFN-beta treatment in blood cells of MS patients^10–13^, a couple of studies suggest that expression of some type I IFN regulated genes may be dysregulated at baseline and correlate with biological response to IFN-beta therapy^14–16^. No study has however provided the systematic analysis of Interferome in treatment-naïve MS subjects at distinct stages of disease. To this end we have exploited the collection of gene expression profiles in response to interferons provided by the Interferome database and mapped Interferome changes in experimental and human MS. Initially we have verified whether IRG transcriptome was enriched in the spinal cord at the acute (20 dpi) and chronic (40 dpi) phases of EAE, in *in vitro* differentiated Th17 cells, and in Th17 and CD4^+^ T cells isolated from EAE CNS compared to controls. Our data clearly demonstrate that CNS neuroinflammation and T cell pathogenicity are characterized by massive changes in Interferome transcription, which is consistent with the known contribution of Type I and Type II interferons to the disease^17^.

The analysis of human PBMC transcriptomes has highlighted 21 concordantly regulated IRG transcripts at all MS stages compared to healthy controls, indicating that the disease is characterized by convergence towards some common IRG targets. Among them, for example we describe transcriptional upregulation of Nod-like receptor family pyrin domain containing 3 (NLRP)-3, a member of the inflammasome complex, and beta-arrestin-1 (ARRB1) a cytosolic protein essential for CD4^+^ T cell survival^18^, two genes whose products support the development of experimental encephalomyelitis^18,19^. In addition, several IRG are dysregulated at distinct MS stages, underlying that differential expression of Interferome is not a completely stable feature throughout the disease. For instance, IRF1 supports EAE expression^20^ and is upregulated in PBMC from SP-MS subjects and not other disease stages. This observation opens to the possibility that immune cells at distinct MS stages display differences in the response to IFN, a hypothesis which deserves further investigation. When verifying the frequency of Interferome dysregulation at distinct MS courses, we have ascertained its significant enrichment in RR-MS, indicating that the relapsing-remitting form of disease is extremely characterized by transcriptional changes in IRG. Notably, 100 IRG dysregulations in RR-MS have been validated in an independent case-control cohort measured with a distinct array platform, and several of them have been already associated with MS pathogenesis. For instance, Caspase (CASP)-1, also known as interleukin 1 beta converting enzyme, regulates inflammatory processes during EAE^21^ and has been already shown to be upregulated in MS blood^22^. In harmony with the published findings, we confirm overexpression of CASP1 transcript in PBMC of RR-MS compared to healthy subjects. Further, S100A9, a member of S100 protein family, is strongly expressed in several inflammatory disorders and promotes the recruitment of immune cells to sites of tissue damage^23^. Its expression increases in the CNS of EAE mice^24^ and our study at 40dpi), in CD4^+^ Th17 T lymphocytes isolated from the CNS of EAE animals and in peripheral blood of RR-MS (our study). Another dysregulated IRG identified in our study is MAF, a transcription factor critical for Th17 T cell differentiation^25^. It is one of the proposed MS risk genes whose expression is upregulated in RR-MS blood^9^, and in pathogenic T lymphocytes from EAE CNS (this study).

Considering the overrepresentation of dysregulated IRG in RR-MS, we have verified whether IFN-beta therapy targets the impaired expression of disease-associated IRG in RR-MS and found that the levels of 53 out of 100 IRG dysregulated in treatment-naïve patients change under IFN-beta treatment. This analysis has led to the identification of several disease-associated transcripts that may serve as markers of biological activity of IFN-beta *in vivo* and potential novel targets for MS treatment. Further *ex vivo* and *in vitro* validations have been performed for ARRB1 and CHP1, whose gene product inhibits the transcriptional activity of Nuclear Factor of Activated T cells^26^. Their transcripts are upregulated in PBMC from treatment-naive RR- MS patients compared to healthy controls. Interestingly, our *in vitro* assays have shown that a short *in vitro* exposure to IFN-beta can induce reversal regulation of ARRB1 and CHP1 transcripts in RR-MS immune cells. Further, we provide evidence that treatment of RR-MS with IFN-beta counterbalances ARRB1 and CHP1 dysregulation present at baseline.

Overall, we demonstrate dysregulation of Interferome in experimental and human MS and describe novel IRG signatures at distinct stages of disease which can be partly targeted by IFN-beta treatment *in vivo*. The correlation between Interferome dysregulation at baseline and clinical response to IFN-beta treatment is missing at this stage, but can be further addressed in future prospective studies. Anyway, our analyses provide the scientific community fundamental knowledge about the specificities of the IRG profiles at distinct MS stages and identify novel potential therapeutic targets in MS.

## Materials and Methods

### Human subjects and peripheral blood mononuclear cell (PBMC) preparation

Investigations were conducted according to the principles expressed in the Declaration of Helsinki and after approval of the Ethics Committee of S. Raffaele and S. Andrea Hospitals, and peripheral blood was drawn after signing of the informed consent. MS subjects were diagnosed according to McDonald criteria^27^, were not suffering from any acute or chronic inflammatory diseases or other autoimmune disorders, and had not started any immunomodulatory therapy for MS unless where indicated. Demographic and clinical data of enrolled patients are listed in Table 1 of ref.^9^ and Supplementary Table 1 of the current manuscript. PBMC were isolated using a discontinuous density gradient (Lympholyte-H, Ficoll-Paque Cederlane). Viable cells were counted by Trypan Blue (Sigma-Aldrich, Milan, Italy) exclusion.

### PBMC stimulation with IFN-beta

For *in vitro* analyses, PBMC were resuspended in RPMI 1640 supplemented with 10% fetal bovine serum (BioWhittaker Europe), penicillin and streptomycin, and L-glutamine (Gibco), and cultured for 16 hours in presence or absence of 50 pM recombinant IFN-β1a (Betaferon, Bayer Pharma).

### Experimental Autoimmune Encephalomyelitis (EAE), collection of CNS tissues and RNA preparation

C57BL/6 female mice were purchased from Charles River Laboratories (Calco, Italy), and housed in the institutional animal house. All procedures involving animals were performed according to guidelines authorized by the local institutional ethical animal committee and the Italian General Direction for Animal Health at the Ministry of Health. EAE was induced in 8 week-old C57BL/6 mice by subcutaneous immunization with 200 µg MOG_35-55_ peptide (Espikem, Florence, Italy) in complete Freund’s adjuvant containing 8 mg/ml Mycobacterium tuberculosis (strain H37Ra; DIFCO, BD, Buccinasco, Italy). Pertussis toxin (500 ng, Sigma-Aldrich) was injected on the day of the immunization and two days later. Animals were monitored daily and scored as previously described^28,29^. At sacrifice EAE and naïve mice were anaesthetized and perfused, spinal cords were isolated and used for transcriptomics studies. RNA was extracted by TRIzol Reagent (Thermo Fischer Scientific, Monza, Italy) following the manufacturer’s protocol.

### Generation and analysis of Microarray Datasets

Spinal cord RNA from 3 EAE mice at day 20 post-immunization (dpi), 3 EAE mice at day 40 post-immunization and 8 age-matched naïve mice were used for the mouse CNS microarray experiment. RNA was checked for integrity at Bioanalyzer 2100 (Agilent, Milan, Italy), converted to cRNA and hybridized on Affymetrix Gene Chip Mouse Genome 430 2.0 arrays expression arrays. Data were deposited at EBI Array express database (accession number: E-MTAB-5255). Affymetrix Gene Chip Operating Software was used to extract the raw data, which were then processed by robust multi-array average (RMA) algorithm in Bioconductor^30^ and keeping genes with multiple probes as individual transcripts. Transcriptomics data relative to mouse CD4 T lymphocytes were retrieved from Gene expression omnibus (GEO) database under the accession number GSE 57098^6^. This dataset comprised three biological samples for each of the following groups: CD4^+^ CD62L^+^ naïve myelin-specific T lymphocytes before (T naïve) and after *in vitro* Th17 differentiation (Th17 iv), CD4^+^ myelin-reactive Th17 T lymphocytes isolated after adoptive transfer from the CNS of EAE mice (Th17eae), CD4^+^ T lymphocytes isolated from the CNS of EAE mice after active immunization (CD4eae)^6^. Mouse CD4 T cell transcriptomic data were generated with Agilent Genome Microarray 4 × 44 K v2 and all the raw data were processed using RMA algorithm in Bioconductor.

The human PBMC microarray datasets analyzed in this study were published in a recent paper of our group^9^ and deposited at EBI Array express database. E-MTAB-4890 dataset was generated by Illumina Human Ref-8 v2 microarrays and contained PBMC transcriptomes of 46 CIS, 52 RR-MS, 23 PP-MS, 21 SP-MS patients, and 40 healthy controls (HC), while E-MTAB-5151 was generated with Affymetrix Gene Chip Human Genome U133 plus 2.0 Arrays and comprised PBMC transcriptomes of 21 RR-MS and 27 healthy subjects. The Genome Studio GX software was used to extract the Illumina raw data, which were then background subtracted by nec method and normalized by cubic spline normalization as implemented in the software. Probes correlating with age in the healthy population were removed. Regarding the Affymetrix dataset, all the necessary pre-processing steps were performed using RMA algorithm present in the Affy package in Bioconductor, and keeping genes with multiple probes as individual transcripts. To assess whether IFN-beta treatment was targeting IRG expression in PBMC from RR-MS, we analyzed part of the PBMC transcriptome dataset published in ref.^8^, generated with Affymetrix Gene Chip Human Genome U133 plus 2.0 and composed of 128 IFN-beta treated and 141 untreated RR-MS subjects. All the raw data were pre-processed using RMA algorithm in the Affy package.

Probes with a mean intensity value lower than 100 in all experimental groups in both mouse and human studies were filtered out. To identify differentially expressed genes (DEG) we performed E-bayes statistics using Limma package in R Bioconductor^30^.

### Collection of IRG and their relative gene expression data

3855 human and 4081 mouse IRG were systematically collected from Interferome (version 2.01, update as of March 2015, http://interferome.its.monash.edu.au/interferome/home.jspx), a manually curated database for interferon regulated genes identified in transcriptomics studies^7^. All the filtered probes in the Illumina PBMC dataset were submitted to DAVID gene ID conversion tool^31^ to retrieve Ensemble and gene symbol identifiers and isolate the probes relative to IRG. As further control, the same probe list was submitted to biomaRt ID conversion package in R Bioconductor^32^. Similarly, for the human/mouse Affymetrix or Agilent datasets IRG were annotated to the corresponding probes by biomaRt ID conversion package in R Bioconductor.

### Frequency of dysregulated Interferome during neuroinflammation

To verify whether MS or EAE transcriptomes were enriched in dysregulated IRG, we measured the frequency of differential expression (DE) in IRG list and in the global transcriptome, and verified whether the frequency of IRG DEG was significantly higher in the IRG list than the expected frequency of dysregulation in a random selection of transcripts from the database by chi-square test with Yates’s correction in Graph Pad. Z score was calculated by Z = r-n ((R/N)/Sqrt (n(R/N) (1-R/N) (1-(n-1/N-1))^33^, where R is the number of dysregulated probes in the global transcriptome, N is the total number of expressed probes in the global transcriptome, r is the number of differentially expressed IRG probes and n is the total number of expressed IRG probes.

### Gene Ontology enrichment analysis

Genecodis program^34^ was used to search for Gene Ontology biological processes enriched in the genelist and selected those terms passing the FDR-corrected p-value threshold of 0.05 and containing three DEG. The database was annotated with approximately 95% of the genes in our gene list.

### RNA extraction, cDNA synthesis, quantitative PCR

DNase I-treated total RNA was purified from *ex vivo* isolated or *in vitro* stimulated PBMC using the RNeasy Mini Kit (Qiagen, Valencia, CA), and reverse-transcribed by the Murine Leukaemia Virus Reverse Transcriptase (Invitrogen Life Technologies, Carlsbad, CA) following the manufacturers’ protocol. Real time PCR for target genes was conducted with TaqMan gene expression assays (Life Technologies, Monza, Italy) corresponding to the gene sequence recognized by the array probe. Thymosin Beta 10 (TMSB10) or Proteasome Subunit Beta 1 (PSMB1) were used as housekeeping genes due to their low coefficient of variation in the Illumina human transcriptomic dataset. Housekeeping genes with expression levels similar to those of the target genes were chosen. Target gene expression data were expressed as percentage of the housekeeping gene as already described^9,35^.

### Statistical analysis

Normality of data distribution was assessed by Kolmogorov–Smirnov statistics. Unpaired t-test (in case of normal distribution) or non-parametric Mann–Whitney U test (in case of non-normal distribution) was performed to compare means between independent groups. Paired t-test was applied to determine the significance of ARRB1 and CHP1 expression differences *in vitro* in absence or presence of IFN-beta stimulation. All the p-values were two-sided and subjected to a significance threshold of 0.05.

### Data Availability

The datasets generated during and/or analysed during the current study are available from the corresponding author on reasonable request.

## Electronic supplementary material

Supplementary information

Supplementary Table 1

Supplementary Table 2

Supplementary table 3

Supplementary table 4
