# Author Correction: Transcriptional dysregulation of Interferome in experimental and human Multiple Sclerosis

**DOI:** 10.1038/s41598-018-24987-8

**Published:** 2018-05-11

**Authors:** Sundararajan Srinivasan, Martina Severa, Fabiana Rizzo, Ramesh Menon, Elena Brini, Rosella Mechelli, Vittorio Martinelli, Paul Hertzog, Marco Salvetti, Roberto Furlan, Gianvito Martino, Giancarlo Comi, Eliana M. Coccia, Cinthia Farina

**Affiliations:** 1 Institute of Experimental Neurology (INSpe), Division of Neuroscience, San Raffaele Scientific Institute, 20132 Milan, Italy; 2 University Vita-Salute San Raffaele, 20132 Milan, Italy; 3 Department of Infectious diseases, Istituto Superiore di Sanità, 00161 Rome, Italy; 4 University “La Sapienza” Rome, Dept. Neurological Sciences, MS center of S. Andrea Hospital, 00185 Rome, Italy; 5 Centre for Innate Immunity and Infectious Diseases, Hudson Institute of Medical Research, Monash University, 3168 Clayton, IS Australia; 6 IRCCS Istituto Neurologico Mediterraneo (INM) Neuromed, 86077 Pozzilli, IS Italy

Correction to: *Scientific Reports* 10.1038/s41598-017-09286-y, published online 21 August 2017

The original version of this Article contained a typographical error in the spelling of the author Eliana M Coccia, which was incorrectly given as Eliana Coccia. This has now been corrected in the PDF and HTML versions of the Article, and in the accompanying Supplementary Material files.

