# Supplementary material for: Transcriptional dysregulation of Interferome in experimental and human Multiple Sclerosis
Source: Sci Rep. 2017 Aug 21;7:8981. doi: 10.1038/s41598-017-09286-y (PMC5566335; doi:10.1038/s41598-017-09286-y)
Supplement: Supplementary file 1 — Supplementary information [file 41598_2017_9286_MOESM1_ESM.pdf]

Transcriptional dysregulation of Interferome in experimental and human Multiple Sclerosis

Sundararajan Srinivasan[a,b], Martina Severa[c], Fabiana Rizzo[c], Ramesh Menon[a], Elena Brini[a], Rosella Mechelli[d], Vittorio Martinelli[a], Paul Hertzog[e], Marco Salvetti[d], Roberto Furlan[a], Gianvito Martino[a,b], Giancarlo Comi[a,b], Eliana M. Coccia[c], Cinthia Farina[a*]
